# Nonlinear Analysis of Sensory Organization Test for Subjects with Unilateral Vestibular Dysfunction

**DOI:** 10.1371/journal.pone.0091230

**Published:** 2014-03-14

**Authors:** Jia-Rong Yeh, Li-Chi Hsu, Chen Lin, Fu-Ling Chang, Men-Tzung Lo

**Affiliations:** 1 Research Center for Adaptive Data Analysis and Center for Dynamical Biomarkers and Translational Medicine, National Central University, Taoyuan, Taiwan; 2 Department of Neurology, Taipei Veterans General Hospital, Taipei, Taiwan; 3 National Yang-Ming University School of Medicine, Taipei, Taiwan; 4 Taiwan Textile Research Institute, New Taipei City, Taiwan; 5 National Yunlin University of Science and Technology Graduate School of Design, Yunlin, Taiwan; University of California, Merced, United States of America

## Abstract

Vestibular disorder is the cause of approximately 50% of dizziness in older people. The vestibular system is a critical postural control mechanism, and posturography analysis is helpful for diagnosing vestibular disorder. In clinical practice, the sensory organization test (SOT) is used to quantify postural control in an upright stance under different test conditions. However, both aging and vestibular disorder cause declines of postural control mechanisms. The aim of this study was to enhance the performance of the SOT using a nonlinear algorithm of empirical mode decomposition (EMD) and to verify the differences of effects caused by aging and/or illnesses benefits to clinical diagnosis. A total of 51 subjects belonging to 3 groups—healthy-young, healthy-elderly and dizzy—were recruited for this study. New dynamic parameters of the SOT were derived from the center of pressure (COP) signals. EMD served as an adaptive filter bank to derive the low- and high-frequency components of the COP. The effects on four ratios of sensory analysis caused by aging and vestibular disorder can be investigated for the specific frequency bands. According to our findings, new SOT parameters derived from the component with the specific frequency band more sensitively reflect the functional condition of vestibular dysfunction. Furthermore, both aging and vestibular dysfunction caused an increase in magnitude for the low-frequency component of the AP-direction COP time series. In summary, the low-frequency fluctuation reflects the stability of postural control, while the high-frequency fluctuation is sensitive to the functional condition of the sensory system. EMD successfully improved the accuracy of SOT measurements in this investigation.

## Introduction

In the USA, 80% of people aged 65 years and older have experienced dizziness and disorder of postural control [1]. Vestibular, somatosensory, and visual inputs contribute to postural control and equilibrium. Of the three sensory channels, vestibular disorder is the cause of dizziness and imbalance in approximately 50% of older people [2]. Posturography is a general term that covers all the techniques used to quantify the relative contributions of these sensory systems to postural control in the upright stance under either static or dynamic conditions [3]. Numerous different protocols have been proposed to differentiate the impairments of the sensory systems for the patient's posture control. The sensory organization test (SOT) is one protocol commonly used in clinical practice. The principle of SOT evaluation is to selectively disrupt the support surface and/or the visual inputs in order to measure the subject's ability for using remaining sensory channels for postural control [4]. The standard comprehensive SOT results comprise equilibrium scores (EQS) of 6 test conditions, 4 ratios of sensory analysis, results of strategy analysis, and reports of center of gravity (COG) alignment. The four ratios of sensory analysis are somatosensory (SOM), visual (VIS), vestibular (VEST), and visual preference (PREF). These analyses are used in conjunction with the equilibrium scores to identify impairments of individual sensory systems.

The SOT is commonly used in studies of populations with different deficits in postural control, such as patients with Parkinson's disease (PD) [5], peripheral vestibular deficits [6], peripheral neuropathy [7], or stroke [8]. Many previous studies using computerized dynamic posturography showed that dysfunctions of different postural control mechanisms cause the changes in posturographic trajectories on specific frequency bands [9]–[12]. In the SOT, the EQS is defined as a nondimensional percentage comparing the individual's peak amplitude of anterio-posterior (AP) sway within the theoretic limits of stability (LOS) in the AP direction. In posturography, the variation of the body's center of gravity (COG) corresponds to the stability of postural control. The increased variations of COG correspond with decreased postural control [13]. Center of pressure (COP) corresponds to the vertical projection of COG under static conditions in which no accelerations are applied [14]. Because measurements of COG variation are technically complicated, cumbersome, costly, and error-prone [15], steadiness can be measured instead by COP variation [16].

Moreover, a nonlinear and non-stationary COP time series involves irregular and unpredictable components [17]. In order to characterize the irregularity and unpredictability of a COP time series, many different nonlinear analyses have been used in previous studies [18], [19], [20]. The approximate entropy (ApEn) [21] represents a typical nonlinear measure that characterizes the dynamics in the output of a complex system. These nonlinear measures represent different profiles of a COP time series. On the other hand, recent research supports [22], [23] that the structure of a COP time series is dependent on the time scale of the observation. Timescale analysis supports a model on the basis of timescale separation and thus simplifies the analysis of the system [24]. Theoretically, many spectral processing techniques based on linear assumptions, such as Fourier and wavelet transform, which are based on predetermined sets of bases, can be used in timescale analysis. The term ‘power leakage’ is always associated with these spectral analysis algorithms [25]. Moreover, slow or irregular trends in a time series can potentially distort spectral analysis and lead to misinterpretations [26]. Empirical mode decomposition (EMD) has been suggested as a good choice of algorithm for extracting the intrinsic trends [27] and for employment as a natural filter bank [28] for a nonlinear and non-stationary time series. A previous study [27] also demonstrated that EMD works as a better algorithm for timescale separation when comparing linear algorithms in the data analysis to nonlinear and non-stationary signals like the COP time series. Therefore, EMD decomposes a COP time series into a limited number of intrinsic mode functions (IMF) [29]. An IMF represents an intrinsic oscillation within a specific timescale range [30], [31]. The syntheses of different IMFs reflect the oscillatory fluctuations on their corresponding timescale ranges; then, the timescale separation of a nonlinear and non-stationary time series can be realized. Root mean square (RMS) can be used to represent the magnitude of the oscillatory fluctuation as the original or as a part of the COP time series.

The aim of the present research was to investigate the magnitude of oscillatory fluctuations of the COP time series on different timescale ranges by EMD. We hypothesized that the magnitudes of oscillatory fluctuations in specific timescale ranges should be associated with the functional decline of sensory systems. An oscillatory fluctuation composed of multiple successive IMFs represents a specific fluctuation within a specific timescale range in a COP time series. The magnitudes of the specific fluctuations are used to replace the EQS and to calculate the ratios of sensory analysis in a SOT evaluation. Consequently, the SOT evaluation can be focused on the desired timescale range.

Based on the results of spectral analysis, three oscillatory fluctuations derived from a COP time series were used in this investigation: the first one covers overall timescales; the second covers high-frequency (HF) fluctuation; and the third one covers low-frequency (LF) fluctuation. According to our observation results, LF fluctuation (<0.5 Hz) of COP contributes the dominant portion of instability of posture control, which reflects the ensemble effects caused by aging and impairment of sensory systems. The increased magnitude of AP-direction LF fluctuation corresponds with the increased instability of postural control. The ratios of sensory analysis derived by the magnitudes of HF fluctuations (>0.5 Hz) are more sensitive to representing the functional declines of sensory systems. The advantages of employing the new sensory analysis in SOT have been established in this study.

## Methodology

### Subjects

The study was approved by the Institutional Review Board of Taipei Veterans' General Hospital, which is a tertiary referral hospital in northern Taipei, Taiwan. The permit number of IRB is VGH-2021-01-040BC. All of the participants were able to provide consent by themselves, and all provided their written consent to participate in this study. The consent procedure was approved by the Institutional Review Board. The 51 subjects recruited to participate in the investigation were divided into three groups: a healthy-young (HY) group, a healthy-elderly (HE) group, and a dizzy patient (DZ) group. The HY group consisted of 23 subjects (19 males, 4 females, mean age: 36.2±5.7, range: 24–48). The HE group was composed of 9 subjects (3 males, 6 females, mean age: 68.6±5.2, range: 62–75). None had any past history of peripheral or central vestibular disorders, other medical or neurological diseases known to cause dizziness, or gait disturbance, and all had normal vestibular function as assessed by classical clinical otoneurologic tests including video-oculographic and caloric tests. There were 19 patients in the DZ group (10 males, 9 females, mean age: 66.7±13.4, range: 40–85). These patients all complained of chronic (>1 month) dizziness and unsteadiness. In addition, they all had unilateral peripheral vestibular hypofunction, as documented by the caloric test. Other than peripheral vestibular hypofunction, central vestibular deficits could also lead to dizziness and unsteadiness. So, patients with central causes of vestibular deficits, such as stroke (brainstem or cerebellum), cerebellar degeneration, vestibular migraine, post-traumatic central disorders, or mixed central and peripheral vestibular deficits were excluded from the current study. Patients who demonstrated abnormal eye-tracking results during video-oculographic tests were also excluded. The study hospital's Institutional Review Board approved this trial, and informed consent was obtained from each participant. The audience can request the raw data by emailing to the corresponding author.

### Sensory organization test (SOT)

Computerized dynamic posturography is a functional test of contribution of the visual, vestibular, and proprioceptive systems to the maintenance of upright posture. The testing provides patients with a combination of present, absent, or distorted visual or proprioceptive cues based on a movable platform support and visual surroundings. In the present study, the SMART Balance Master (NeuroCom, Clakamas, OR, USA) platform posturography was used for evaluation, where the protocol calls for both a sensory organization test (SOT) and motor coordination subsets. Only the SOT segment was used in the current experiment.

**Figure 1:**
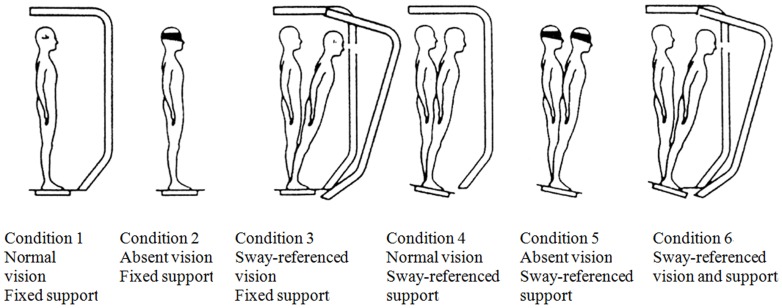
Conditions of sensory organization test (SOT).

The SOT protocol objectively identifies abnormalities in the patient's use of the three sensory systems that contribute to postural control: somatosensory, visual, and vestibular. By controlling the usefulness of the sensory (visual and proprioceptive) information through sway referencing and/or eyes open/closed conditions, the SOT protocol systematically eliminates useful visual and/or support surface information and creates sensory conflict situations. Six test conditions for the SOT were illustrated, as shown in Figure 1: 1. eyes open with fixed surrounding and support. 2. eyes closed with fixed support. 3. sway-referenced surroundings with fixed support. 4. eyes open with fixed surroundings and sway-referenced support. 5. eyes closed with sway-referenced support. 6. sway-referenced surroundings and support. EQS was measured as a nondimensional percentage comparing an individual's peak amplitude of AP sway with the theoretic limits of stability (LOS). The sensory analysis aims to interpret the functional compensations in the sensory systems. The SOM ratio is defined as the ratio between EQS of conditions 1 and 2. Patients with lower SOM have impaired ability to use the available SOM input. The VIS ratio is defined as the ratio between the EQS of conditions 1 and 4. Patients with lower VIS have an impaired ability to use the remaining visual input. The VEST ratio is defined as the ratio between the EQS of conditions 1 and 5. Patients with lower VEST ratios have an impaired ability to use the remaining vestibular input. The PREF ratio is defined as the ratio comparing the sum of the EQS on conditions 2 and 3 with the sum of the EQS on conditions 5 and 6. A low PREF ratio indicates that the patient's balancing ability declines with conflicting visual surroundings. Each subject undertook 3 successful trials to remain standing without falling, under 6 test conditions. SOT reports were automatically generated by the SMART Balance Master. The COP time series was then derived from the raw recordings of the force platform at a sampling rate of 100 Hz. The duration of each trail was 20 seconds, and the data length includes 2000 samples.

### Empirical mode decomposition (EMD)

EMD suggests the mean of upper and lower envelopes, which reflect the highest level of local trends, to derive the local oscillation with the highest frequency band. A decomposed local oscillation is named as an intrinsic mode function (IMF). The signal is considered to consist of many levels of local oscillations and the final monotonic trend. A residue is considered to be a trend, which consists of all un-decomposed local oscillations (i.e., IMFs) and the final monotonic trend.

To a signal *x(t)*, the proposed algorithm of EMD is suggested as follows [29]:

1. Connect the local maxima (respective minima) to derive the upper (respective lower) envelop using cubic spline.
2. Derive the mean of envelope, *m(t)*, by averaging the upper and lower envelopes.
3. Derive the temporary local oscillation *h(t) = x(t)−m(t)*.
4. Repeat steps 1 to 3 on the temporary local oscillation *h(t)* until *m(t)* is close to zero. Then, *h(t)* is an IMF noted as *c(t)*.
5. Compute the residue *r(t) = x(t)−c(t)*.
6. Repeat the steps from 1 to 5, using *r(t)* for *x(t)*, to generate the next IMF and residue.

Therefore, the original signal x(t) can be reconstructed by the following formula:
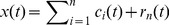
(1)where *c_i_(t)* is the *i*th IMF (i.e., local oscillation) and *r_n_(t)* is the *n*th residue (i.e., local trend).

EMD was proved as an adaptive filter bank for nonlinear and non-stationary signals [28]. IMFs decomposed by EMD are nonlinear and non-stationary components with narrow frequency-bands. A synthesis of several successive IMFs reflects the oscillatory fluctuation within a specific timescale range as a part of the original time series. The magnitude of a varying quantity, such as the original COP time series and the oscillatory fluctuation within a specific timescale range as a part of COP, can be easily quantified using the value of root mean square (RMS).

### New ratios of sensory analysis derived from the magnitude of fluctuation

As mentioned above, the RMS value was used to quantify the magnitude of an oscillatory fluctuation—such as the original or a part of COP time series—in this study. Thus, we can derive the magnitudes for the original COP time series and different syntheses of several successive IMFs according to the timescale ranges in which we were interested. Furthermore, the ratios of sensory analysis can be redefined using the magnitudes of the oscillatory fluctuations:
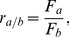
(2)where 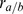 represents a ratio of sensory analysis derived by the magnitudes of the oscillatory fluctuations; and *F_a_* and *F_b_* are the magnitudes of oscillatory fluctuations for test conditions *a* and *b*.

Since the EQS inversely corresponds to the magnitude of COP time series [13], the ratio values derived using the magnitudes of fluctuations represent inverse meanings to those derived using EQS.

### Statistical analysis

In this study, the non-parametric statistics were used according to the consideration of population distributions. Moreover, since there are three groups in this investigation, a Kruskal–Wallis (K-W) one-way analysis of variance by ranks was used for testing whether samples originate from the same distribution. The statistical results of the K-W tests were shown in the K and *p* values. Furthermore, the Kolmogorov–Smirnov (K–S) test was used to verify the statistical difference between two populations in a paired test. The statistical results of the K-S test were shown in the degree of freedom, statistical value, and *p* value. The statistical value of a K-S test is the suprenum of distance between the cumulative distribution functions of two groups. The level of significance for the K-W test was set at *p*<0.05/3; and for the K–S test, the level was set at *p<*0.05.

## Results

### Results of the original SOT report

Statistical analyses of the original SOT results are shown in Table 1. The EQS of the DZ group is only significantly different from that of the HE group under condition 1, (*KS(27) = 0.596, p<0.01*). No ratio of sensory analysis demonstrated a significant difference between the HE and DZ groups. Values of the EQS for HY are statistically different from those for HY under conditions 1 (*KS(30) = 0.564, p = 0.01*), 3 *(KS(30) = 0.501, p<0.05)*, 4 *(KS(30) = 0.498, p<0.05)*, and 5 *(KS(30) = 0.567, p = 0.01)*. According to the results of sensory analysis, the ratios of VIS and VEST for HY are statistically higher than those for DZ, *(KS(39) = 0.568, p = 0.01)* for VIS, and *(KS(39) = 0.465, p<0.05)* for VEST. According to the results of the original SOT reports, both aging and vestibular dysfunction caused the loss of balancing ability. The differences between HE and DZ are statistically insignificant.

**Table 1:** Statistical results of the original SOT reports comprising equilibrium scores (EQS) of 6 test conditions and 4 ratios of sensory analysis.

parameter	Healthy young	Dizzy	Healthy elderly	Statistical results
EQS of C1	95.67±1.15^ee^	95.44±1.35^ee^	93.33±1.59^yy,dd^	5.43(0.066)
EQS of C2	92.06±1.96	92.18±3.01	90.54±2.16	1.20(0.547)
EQS of C3	91.87±3.89^e^	89.33±4.83	87.38±5.90^y^	3.84(0.146)
EQS of C4	87.28±4.58^d^^,^ e	82.07±6.35^y^	82.08±6.77^y^	5.06(0.080)
EQS of C5	67.65±9.03^d^ ^,ee^	56.56±8.42^y^	56.25±14.32^yy^	5.26(0.072)
EQS of C6	70.39±13.80^d^	55.07±15.23^y^	62.96±9.74	7.97(0.019)
SOM	.962±.017	.966±.026	.970±.017	1.07(0.585)
VIS	.912±.047^d^	.860±.060^y^	.879±.064	4.80(0.090)
VEST	.707±.093^dd,^ e	.593±.089^yy^	.601±.145^y^	5.43(0.066)
PREF	1.018±.086	.972±.100	1.027±.048	3.32(0.190)

“y” denotes statistically significant difference when compared with the HY group.

“d” denotes statistically significant difference when compared with the DZ group.

“e” denotes statistically significant difference when compared with the HE group.

Single character denotes with P-value<0.05; double character denotes P-value<0.01.

Values shown in mean ± standard deviation. The statistical results of Kruskal-Wallis test are shown in K value (P value).

### Relationship between EQS and magnitudes of COP time series on both medio-lateral (ML) and/or AP directions

**Figure 2:**
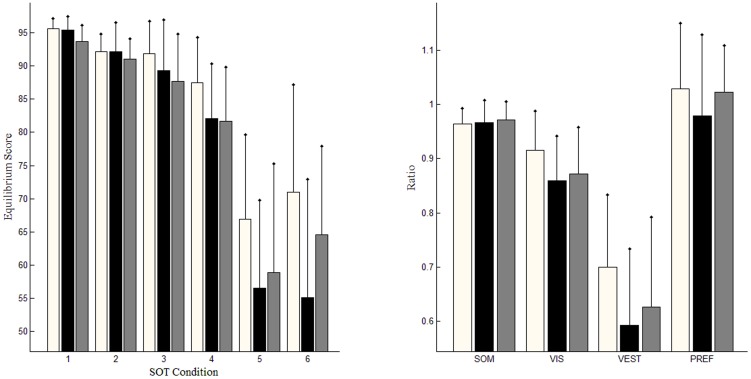
Correlations between EQS and fluctuations of CoP time series in ML and AP directions, (a) compared with fluctuations in ML direction; (b) compared with fluctuations in AP direction. “o” represents samples of the HY group; “.” represents samples of the DZ group; and “x” represents samples of the HE groups.

There are two dimensions in which to depict the moving directions of COP: AP and ML. In the SOT, the EQS is defined as the percentage comparing peak amplitude of AP sway with the LOS. In this study, the magnitudes of oscillatory fluctuations derived from the COP time series in the AP and ML directions with strong correlations with EQS are considered. The linear correlation between the EQS and the magnitudes of COP time series was evaluated using the Pearson product-moment correlation coefficient [32]. Figure 2 shows the relationships between the EQS and the magnitude of the COP time series in either ML or AP directions. The Pearson correlation coefficient between the EQS and the magnitude of the COP time series in the AP direction is −0.9736, which reflects an excellently negative correlation between them. The Pearson correlation coefficient between the EQS and the magnitude of the COP time series in the ML direction is significantly lower than that between the EQS and the magnitude of the COP time series in the AP direction.

### Spectra of AP-direction COP time series

**Figure 3:**
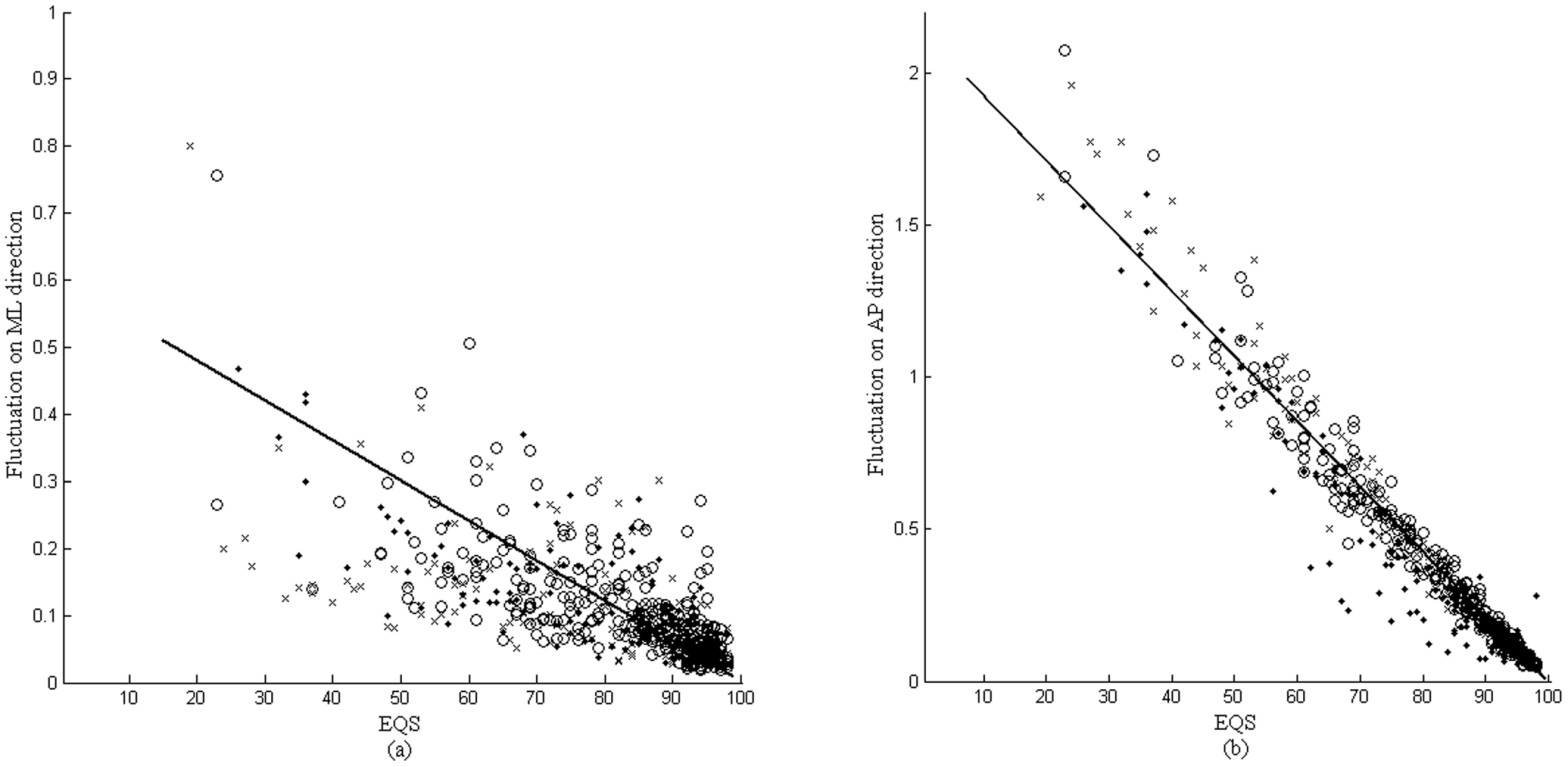
Fourier spectra of AP-direction CoP time series for different groups on 6 SOT test conditions. Arrows pointed out the spectral differences in comparing DZ group with HY and HE groups.

**Figure 4:**
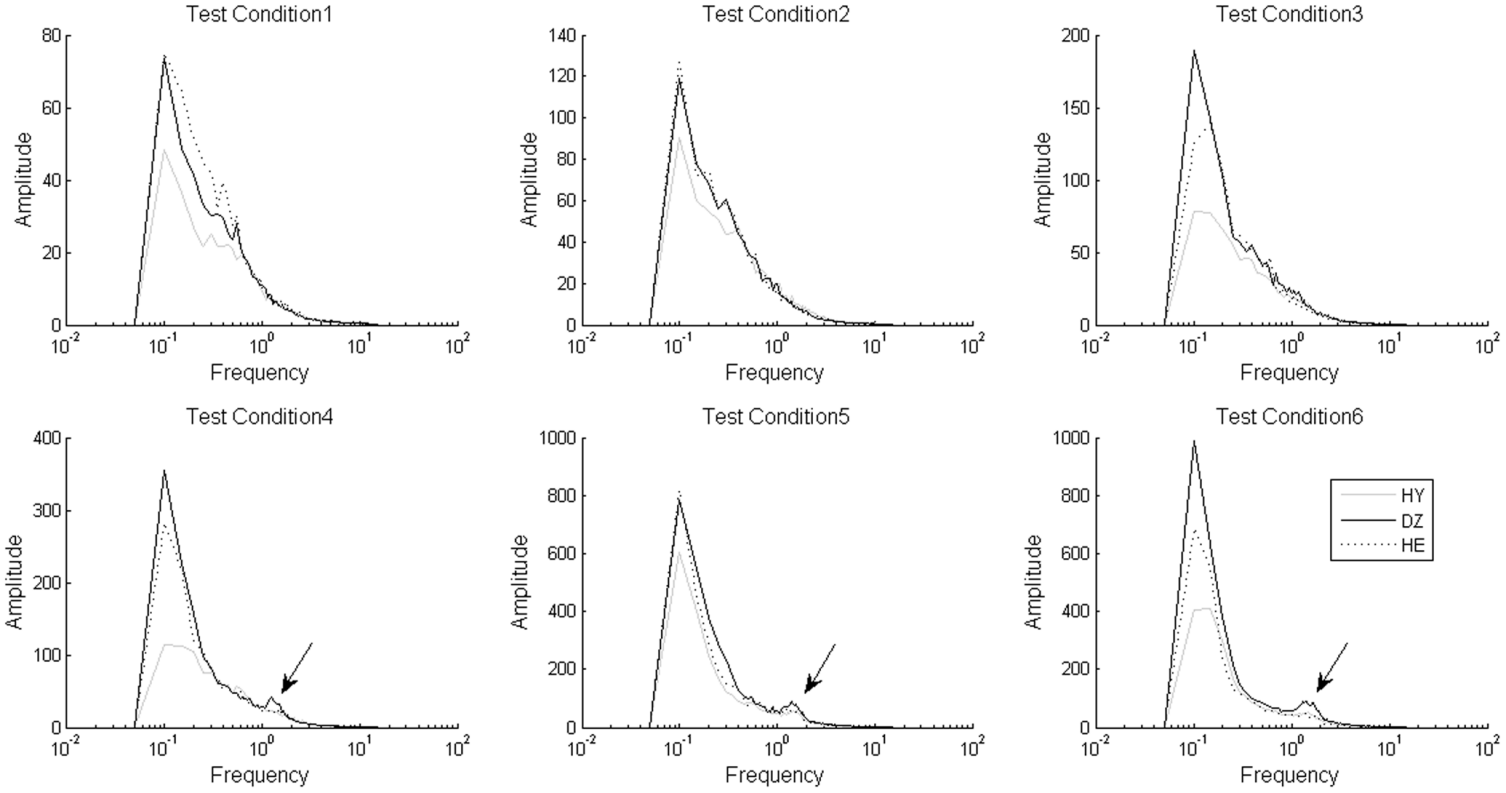
Fourier spectra of IMF 4 of the AP-direction COP time series for DE group in the SOT under 6 test conditions.

For the purpose of conducting an appropriate timescale separation, the traditional Fourier transform was used to examine the frequency-domain characteristics of the COP time series. The AP-direction COP time series was transformed to Fourier spectra as shown in Figure 3. There are peaks, indicated by the arrows, within the frequency band from 1 to 2 Hz that were observed in the spectra for the DZ group under test conditions 4, 5, and 6. These peaks are insignificant in the spectra for the HY and HE groups. For the purpose of checking the findings of the spectral analysis, the EMD decomposed the first 6 IMFs from the COP time series. IMF 4 represents an intrinsic component of the COP with a frequency band of around 1 to 2 Hz, similar to the frequency band indicated by the arrows in Figure 4. Based on the findings of the spectral analysis, we divided the COP time series into two partitions: an HF fluctuation as a synthesis of the first 4 IMFs, and an LF fluctuation as the residual excluding the first 4 IMFs.

### The new SOT results using the magnitudes of LF and HF fluctuations

In sensory analysis using the magnitudes of the oscillatory fluctuations extracted by EMD, high ratios imply an impaired ability to use the corresponding sensory channel. Table 2 shows the results of the new SOT analysis using the magnitude of HF fluctuation. The magnitude of AP-direction HF fluctuations for the DZ group under test conditions 5 and 6 are stronger than those for the other two groups, both for condition 5: *KW(2,49) = 8.87, p = 0.012* and condition 6: *KW(2,49) = 19.62, p<0.0001*. Moreover, VIS, VEST, and PREF ratios using HF fluctuations of the AP-direction COP time series for the DZ group were significantly higher than those for the HY and HE groups, as shown in Table 2, especially the VEST ratio *(KW(2,48) = 11.16, p = 0.004)*. This result implies that the VEST ratio using AP-direction HF fluctuations actually reflects the functional deficit of the vestibular system rather than an aging effect, which is an important finding of our study. The statistical results using HF fluctuations of ML-direction COP time series show no significant differences.

**Table 2:** Statistical results of the new SOT reports using HF fluctuation F_hf_.

Parameter	Healthy young	Dizzy	Healthy elderly	Statistical Results
AP-direction				
F_hf_ of C1	.026±.015	.026±.012	.029±.013	1.14(0.565)
F_hf_ of C2	.049±.033	.041±.021	.040±.017	0.45(0.799)
F_hf_ of C3	.054±.053	.058±.043	.045±.018	1.37(0.505)
F_hf_ of C4	.085±.060	.103±.062^e^	.079±.031^d^	1.85(0.396)
F_hf_ of C5	.173±.082^dd^	.242±.120^yy^	.192±.102	8.87(0.012)
F_hf_ of C6	.172±.105^dd^	.288±.140^yy,ee^	.153±.060^dd^	19.62(<0.0001)
SOM	1.86±0.61^e^	1.65±0.59	1.39±0.45^y^	5.17(0.075)
VIS	3.38±1.17	4.10±1.34^e^	2.87±0.75^d^	7.89(0.019)
VEST	7.05±1.88^dd^	10.21±3.61^yy,^ e	6.96±2.94^d^	11.16(0.004)
PREF	1.01±0.29^d^	1.30±0.48^y^^,^ e	0.90±0.16^d^	10.83(0.004)
ML-direction				
F_hf_ of C1	.016±.010	.016±.008	.016±.006	1.37(0.504)
F_hf_ of C2	.020±.012	.017±.009	.040±.007	0.52(0.771)
F_hf_ of C3	.021±.015	.022±.016	.022±.015	0.68(0.712)
F_hf_ of C4	.029±.021	.029±.016	.030±.013	1.33(0.515)
F_hf_ of C5	.038±.082	.044±.023	.041±.023	3.20(0.201)
F_hf_ of C6	.065±.119^d^	.065±.057^y^^,^ e	.039±.014^d^	7.67(0.021)
SOM	1.42±0.75	1.10±0.30	1.04±0.31	3.28(0.194)
VIS	1.88±0.74	1.91±0.78	1.88±0.64	0.004(1.00)
VEST	2.58±0.97	2.96±1.24	2.50±0.96	1.23(0.540)
PREF	1.60±2.23^e^	1.54±0.88^e^	1.21±0.77^y^^,^ d	4.34(0.114)

“y” denotes statistically significant difference when compared with the HY group.

“d” denotes statistically significant difference when compared with the DZ group.

“e” denotes statistically significant difference when compared with the HE group.

Single character denotes with P-value<0.05; double character denotes P-value<0.01.

Values shown in mean ± standard deviation. The statistical results of Kruskal-Wallis test are shown in K value (P value).

Furthermore, Table 3 shows the results of the SOT using the magnitudes of LF fluctuations. It is interesting that the magnitudes of AP-direction LF fluctuations under test conditions 1, 3, and 4 significantly reflect differences when comparing the HY group with the other two groups, as indicated in Table 3. The magnitudes of AP-direction LF fluctuation for the HY group are significantly smaller than those for the other two groups, as indicated in Table 3. These results are similar to the results shown in the standard SOT reports using the EQS. Moreover, the VEST ratio derived from the AP-direction LF fluctuations didn't reflect the impairment of vestibular sensory input for the DZ group when compared to the HY and HE groups. The statistical results using LF fluctuations of the ML-direction COP time series show no significant differences, as indicated in Table 3.

**Table 3:** Statistical results of the new SOT reports using LF fluctuation F_lf_.

Parameter	Healthy young	Dizzy	Healthy elderly	Statistical results
AP-direction				
F_lf_ of C1	.079±.027^d^ ^,ee^	.105±.071^y^	.121±.053^yy^	8.93(0.012)
F_lf_ of C2	.142±.052^e^	.170±.129	.171±.069^y^	1.80(0.407)
F_lf_ of C3	.147±.082^d^^,^ e	.251±.244^y^	.210±.101^y^	6.90(0.032)
F_lf_ of C4	.220±.114^dd,ee^	.391±.271^yy^	.335±.178^yy^	10.67(0.005)
F_lf_ of C5	.647±.277^dd^	.921±.375^yy^	.818±.345	11.14(0.004)
F_lf_ of C6	.548±.350^dd,^ e	1.016±.464^yy,^ e	.686±.342^y^^,^ d	18.91(<0.0001)
SOM	1.88±0.53^e^	1.65±0.46	1.49±0.43^y^	4.25(0.126)
VIS	2.92±1.96	3.89±1.81	2.79±1.04	4.32(0.116)
VEST	8.73±4.13	9.91±4.04	7.17±2.79	4.13(0.127)
PREF	0.92±0.32^d^	1.18±0.34^y^	0.97±0.19	8.51(0.014)
ML-direction				
F_lf_ of C1	.050±.035	.054±.032	.060±.035	4.20(0.122)
F_lf_ of C2	.055±.038	.063±.046	.075±.042	2.54(0.280)
F_lf_ of C3	.061±.032	.076±.066	.084±.046	2.00(0.369)
F_lf_ of C4	.089±.049	.098±.060	.121±.059	2.03(0.363)
F_lf_ of C5	.121±.057	.156±.076	.171±.107	4.42(0.110)
F_lf_ of C6	.165±.139	.179±.113	.173±.067	3.07(0.215)
SOM	1.28±0.87	1.21±0.53	1.23±0.48	0.04(0.980)
VIS	2.05±0.86	1.92±0.86	2.00±0.67	0.28(0.870)
VEST	2.73±1.07	3.17±1.23	2.83±1.22	0.74(0.692)
PREF	1.40±1.02	1.25±0.56	1.23±0.64	0.61(0.736)

“y” denotes statistically significant difference when compared with the HY group.

“d” denotes statistically significant difference when compared with the DZ group.

“e” denotes statistically significant difference when compared with the HE group.

Single character denotes P-value<0.05; double character denotes P-value<0.01.

Values shown in mean ± standard deviation. The statistical results of Kruskal-Wallis test are shown in K value (P value).

## Discussion

In this study, we redefined the items in the SOT reports using the magnitudes of oscillatory fluctuations on different frequency bands. According to our results, aging causes an increase of LF fluctuations in the AP direction under conditions 1, 2, and 3, as well as both LF and HF fluctuations of the AP-direction COP time series in the SOM ratios of sensory analysis. The SOT results using magnitudes of AP-direction COP time series under conditions 4, 5, and 6 reflect the functional declines of the sensory systems. Both the EQS and magnitudes of AP-direction LF fluctuations represent the ensemble loss of stability of postural control caused by both aging and dysfunction of the vestibular system. On the other hand, magnitudes of AP-direction HF fluctuations reflect a strong correlation with the functional decline of the vestibular system when comparing the VEST ratios of the DZ group to those of the HY and HE groups.

Theoretically, EMD decomposes a nonlinear and non-stationary COP time series to a set of IMFs. An IMF represents a narrow-band oscillatory mode as a counterpart to the simple harmonic function, and it can have variable amplitude and frequency along the time axis. Moreover, IMFs are theoretically orthogonal to each other, which implies that these intrinsic components are independent to each other without coupling. Therefore, EMD can play an important role as an adaptive filter bank for extracting the oscillatory fluctuations on their corresponding frequency bands. The decomposition of EMD is totally adaptive to the nature of a nonlinear and non-stationary signal.

During the last decade, EMD had been used in many studies of COP time series analysis for different purposes. As a detrending tool, EMD was used to remove the trend from a COP time series for enhancing the performance of complexity analysis [35]. As a filter bank, the cumulative sums of the IMFs decomposed from a COP time series were quantified using sample entropy [36] or central tendency measure (CTM) [37]. As a tool for component extraction, EMD was used to detect the specific postural responses to small amplitude AP-direction sinusoidal translations of varying frequencies [38]. Furthermore, the modified method of EMD integrates both ML- and AP-direction COP time series as the bivariate EMD for postural stability analysis [39]. In these studies, EMD actually worked well in different roles in nonlinear and non-stationary signal processing.

On the other hand, many different nonlinear analysis methods were used to characterize the dynamics profiles of the COP time series in the SOT, such as approximate entropy (ApEn) [13]–[20]. The dynamics of a COP time series should be timescale-dependent. In this study, we hypothesized that the oscillatory fluctuations of the COP time series on different timescale ranges in the SOT can reflect the functional conditions of sensory organization and the ensemble stability of postural control, respectively. The function of timescale separation by EMD was associated with sensory analysis as a new approach to the SOT.

It should be mentioned that a subject experiencing dysfunction of the bilateral peripheral vestibular sensory channels usually fell down under SOT test conditions 5 and 6 [4]. In this study, dizzy subjects were patients with unilateral peripheral vestibular hypofunction. The patient suffering unilateral vestibular loss shifts sensory dependence to the remaining vestibular function [33], [34]. During the test, DZ subjects could remain standing under SOT conditions 5 and 6.

Physiologically, the major difference between the DZ group and the other two groups lies in the presence or absence of vestibular hypofunction. The new VEST ratio using the magnitude of AP-direction HF fluctuations revealed significant differences in comparing the DZ group with the other two groups. For both the HE and DZ groups, the magnitude of AP-direction LF fluctuation was significantly larger than that of the HY groups under all SOT test conditions, as shown in Table 3. No significant difference between HE and DZ groups can be found in the SOT results using AP-direction LF fluctuation. The question is: How can this happen? In the clinical practice of SOT tests, subjects with vestibular hypofunction always fall under test conditions 5 and 6. It is important for diagnosing a subject with vestibular hypofunction by SOT. However, all subjects recruited in this study were able to keep standing upright under all test conditions. It is difficult to differentiate aging subjects and subjects with vestibular hypofunction in either the SOT results using the EQS or AP-direction LF fluctuation. This implied an important finding of this study: that the ratios of sensory analysis using AP-direction HF fluctuation can actually differentiate aging subjects from subjects with vestibular hypofunction in SOT.

In summary, we present an approach to investigating the oscillatory fluctuations of the COP time series in different frequency bands with an innovative EMD algorithm. Our results show that the magnitude of AP-direction HF fluctuation of the COP time series is more sensitive in pinpointing the functional conditions of the vestibular system. This is a pilot study for investigating the fluctuations on localized scales by an adaptive nonlinear filter bank. The new approach using magnitude of oscillatory fluctuations of particular timescale ranges actually improved the results of the SOT. However, there are still critical limitations to this study: (1) the sensory ratios can be quantified only for subjects who are able to remain standing under test conditions 5 and 6; (2) the reproducibility of the SOT, which can be represented by the coefficient of variation (CV), is still low. In practical applications, the problem of reproducibility can be diminished by performing several trials for each test condition.

Furthermore, the variances of the magnitudes and the ratios for the oscillatory fluctuations derived from the ML-direction COP time series are large, which resulted in the statistical results being insignificant. Therefore, AP-direction COP time series is a better choice than ML-direction COP time series for quantifying the sway of postural control. Nonlinear measures may be different choices for evaluating the dynamics of ML-direction COP time series in future works.

Finally, according to our findings, the magnitude of AP-direction HF fluctuation seems to sensitively correspond with the functions of sensory organization, and the magnitude of AP-direction LF fluctuation corresponds with the instability of postural control. The increased magnitude of AP-direction LF fluctuation similar to the EQS corresponds with decreased postural control. Therefore, the increased magnitude of AP-direction LF fluctuation corresponds with a high risk of falling. Moreover, we suggest that the ratios of sensory analysis should be calculated using the magnitudes of HF fluctuation of AP-direction COP time series, but not the original or the magnitude of LF fluctuation.
